# Major Factors Affecting the Live Birth Rate After Frozen Embryo Transfer Among Young Women

**DOI:** 10.3389/fmed.2020.00094

**Published:** 2020-03-24

**Authors:** Ye Pan, Guimin Hao, Qiumin Wang, Hong Liu, Ze Wang, Qi Jiang, Yuhua Shi, Zi-Jiang Chen

**Affiliations:** ^1^Center for Reproductive Medicine, Shandong University, Jinan, China; ^2^National Research Center for Assisted Reproductive Technology and Reproductive Genetics, Shandong University, Jinan, China; ^3^Key Laboratory of Reproductive Endocrinology of Ministry of Education, Shandong University, Jinan, China; ^4^Shandong Provincial Clinical Medicine Research Center for Reproductive Health, Shandong University, Jinan, China; ^5^Department of Reproductive Medicine, The Second Hospital of Hebei Medical University, Shijiazhuang, China

**Keywords:** duration of infertility, endometrial thickness, frozen embryo transfer, factors, live birth rate

## Abstract

In recent years, the freeze-all strategy has been widely adopted and applied. However, with the exception of age, the factors that affect the outcomes of frozen embryo transfer are still unclear. Therefore, the identification and mitigation of factors that influence the live birth rate after frozen embryo transfer is a good way to increase the “take-home-baby” rate of frozen embryo transfer. The objective of this study was to identify factors affecting the live birth rate after cleavage-stage frozen embryo transfer in young ovulatory women. This was a secondary analysis from a previously published multicenter randomized controlled trial (ChiCTR-IOR-14005406) that was originally designed to compare the live birth rate and perinatal complications after fresh embryo transfer to those after frozen embryo transfer among ovulatory women. This study was carried out using a portion of the data from the original randomized controlled trial, which included 917 young women who underwent cleavage-stage frozen embryo transfer. The 16 clinical candidate variables potentially affecting the live birth rate after frozen embryo transfer were analyzed. Univariable analysis and multivariable analysis were performed to assess the relationship between predictive factors and outcomes, with the aim of identifying independent predictors of live birth after frozen embryo transfer. In this study, the live birth rate was 53.0% (486/917). Three independent predictors were ultimately identified as the main factors affecting the live birth rate of ovulatory young women. Infertility duration [odds ratio (OR): 0.933, 95% confidence interval (CI): 0.876–0.995, *p* = 0.033], endometrial thickness before frozen embryo transfer (OR: 3.375, 95% CI: 1.556–7.321 *p* = 0.002), and the number of embryos transferred (OR: 2.653, 95% CI:1.226–5,743, *p* = 0.013) were the major factors contributing to the live birth rate after cleavage-stage frozen embryo transfer among young women. The cut-off point for infertility duration was 4.5 years, and the cut-off point for endometrial thickness was 0.89 cm. Infertility duration, endometrial thickness and number of embryos transferred might affect the live birth rate after frozen embryo transfer among young women. This result could help inform clinical decisions and counseling to increase the live birth rate after frozen embryo transfer among young women.

## Introduction

In 1983, the first successful pregnancy after frozen embryo transfer (FET) was reported, and since then, FET has become an important procedure for infertility treatment (1, 2). However, compared with that of fresh embryo transfer, the pregnancy outcome of FET is controversial. To verify the effects of freezing embryos on pregnancy outcomes, many studies have compared the pregnancy outcomes of FET with those of fresh embryo transfer (3–6). Several studies have reported no significant difference between the live birth rate (LBR) after FET and that after fresh embryo transfer in ovulatory women (3, 4). Other studies reported that the LBR after FET was better than that of fresh embryo transfer (5, 6). Additionally, FET provides many advantages the ability to achieve maximum oocyte retrieval by storing additional viable embryos (7), reduce the incidence of ovarian hyperstimulation syndrome, to decrease the multiple pregnancy risk by reducing the number of transferred embryos (8), test the embryos of couples with a genetic disease (9), and arrange opportunities for unsynchronized embryo donations (10). Therefore, although fresh embryo transfer is still routine, in recent years, the freeze-all strategy has been widely adopted and applied. Identifying and influencing the factors that influence FET outcomes can help to increase the “take-home-baby” rate of FET. Hence, the focus of research has recently shifted toward identifying relevant factors that affect FET outcomes. Various clinical factors, including female age, body mass index (BMI), infertility duration, infertility etiology, protocol type, endometrial preparation regimen for FET, and so on, are thought to affect FET outcomes (11, 12). Age is a well-known factor that affects the LBR after FET. However, other factors that could affect the outcome of FET are still unclear. Although the number of older women with infertility has increased annually, young women with infertility still constitute most of the infertility population. The factors that affect the FET outcomes of young women are common concerns among physicians. Hence, we chose young patients (age ≤ 35 years) as the target population to identify the factors affecting the LBR after FET in young women.

We recently completed a large multicenter randomized controlled trial (RCT) that compared the pregnancy outcome of fresh embryo transfer to that of FET in ovulatory women (3). The results of this RCT indicated no significant difference in the LBR between fresh embryo transfer and FET. The current study is a secondary analysis of the FET portion of the RCT. The purpose of this study was to determine the factors affecting the outcome of FET, increase the LBR, and provide information for clinical decisions and consultations.

## Materials and Methods

### Study Population

This secondary analysis data was derived from a previously published multicenter RCT in China. The original study was carried out by 20 participating clinical sites in China and recruited 2157 ovulatory women with infertility who underwent their first *in vitro* fertilization (IVF) or intracytoplasmic sperm injection (ICSI) cycle from March 2015 to November 2015. The original study obtained ethics approval from Ethics Committee at the Center for Reproductive Medicine, Shandong Provincial Hospital Affiliated to Shandong University ([2014] No. 18) and each participating center. All patients signed informed consent forms before inclusion in the study and were identified by the participant number only. We included only 917 participants who ultimately underwent the cleavage-stage frozen embryo transfer procedure (excluding those with missing data) (Supplemental Figure 1). The details of the administration and main outcome of the RCT have been reported (3).

The inclusion criteria were as follows: ovulatory women, aged 25–35 years, who had infertility for more than 1 year, were diagnosed with tubal factor or male factor infertility, and had undergone their first *in vitro* fertilization (IVF) or intracytoplasmic sperm injection (ICSI) cycle. The exclusion criteria were unilateral oophorectomy history, polycystic ovary syndrome (PCOS) history, uterine abnormality, abnormal parental karyotypes, recurrent spontaneous abortion, or chronic medical conditions that contraindicated pregnancy.

### Study Procedures

All participants underwent a standard ovarian stimulation protocol and luteal phase support protocol according to a routine method. The complete details of the trial design and the main outcomes have been previously reported (3). Controlled ovarian hyperstimulation and oocyte retrieval protocols have been extensively described. Treatment with recombinant follicle-stimulating hormone (rFSH, Puregon, MSD, Ravensburg, Germany) 75~225 IU was initiated on days 1–3 of the menstrual cycle and continued for 5 days. Then, the rFSH dose was adjusted, and human menopausal gonadotropin (HMG, Menopur, Ferring, Switzerland) was added to the regimen according to the patient's ovarian response at the discretion of the local investigators. Gonadotropin-releasing hormone antagonist (Ganirelix, Orgalutran, MSD, Ravensburg, Germany) 0.25 mg was started when the leading follicle was ≥12 mm. Human chorionic gonadotropin (HCG 4,000–8,000 IU) was administered when at least 3 follicles were ≥18 mm, and follicle aspiration was performed 34–36 h later. After follicular aspiration, IVF/ICSI or rescue ICSI was performed. The embryos were assessed by the number and regularity of the blastomeres and the percentage of fragmentation. In the primary RCT, participants were randomly divided into a fresh embryo group and a frozen embryo group. In this analysis, we included only women who had ultimately transferred D3 frozen embryos and excluded those who were lost to follow-up and those with missing data. All the embryos of these patients were cryopreserved by vitrification. After the oocyte was aspirated, endometrial preparation began as early as the next menstrual cycle. For endometrial preparation, a natural or artificial cycle was chosen by local physicians. The natural cycle was the first choice. If the natural ovulation cycle was canceled, an artificial cycle was used in next the cycle. For natural cycles, ovulation in the participants was monitored by ultrasound. Two embryos were transferred 3 days after ovulation. For luteal phase support, after ovulation, participants started oral dydrogesterone 10 mg twice daily until 10 weeks of gestation. For artificial cycles, the participants were given 4–8 mg estradiol valerate per day, and then endometrial thickness was monitored by ultrasound. When the endometrial thickness was ≥7 mm, two embryos were transferred according to their cryopreservation stages. The participants began their use of vaginal progesterone gel 90 mg/d and oral dydrogesterone 10 mg twice daily. When the patient became pregnant, estradiol (E2) use was gradually stopped. Vaginal progesterone gel use continued until the fetal heartbeat was heard, and oral dydrogesterone was stopped at 10 weeks of gestation. All pregnancies were included in the follow-up assessment until delivery.

### Statistical Analysis

Live birth was the primary endpoint of the study. Sixteen candidate variables that may affect the LBR after FET were selected according to clinical experience and the literature. First, descriptive statistics and univariate analyses were performed. Continuous variables were summarized as the mean ± SD and compared between the live birth group and no live birth group by *t*-test. Qualitative variables were summarized with percentages and compared between live birth cycles and no live birth cycles by the chi-square test or Fisher's exact test. When the individual expected count was <5, we used Fisher's exact test. Then, univariate and multivariate logistic regression were used to assess the relationships between predictive factors and outcomes. Clinically relevant predictive variables and univariable analysis covariates with *p* < 0.15 were included in the multivariable analysis. Finally, 8 predictors were analyzed in the multivariable analysis, including the infertility duration, causes of infertility, endometrial thickness, endometrial preparation regimen for FET, LH level, number of embryos transferred, number of top-quality embryos, and oocyte fertilization method. To avoid collinearity, the variance inflation factor (VIF) was used to detect the presence of collinearity between two or more independent variables in this multivariable model. No collinearity was detected among variables. In the multivariate logistic regression analysis, independent factors were determined by a backward variable selection approach in which the least significant variable was removed one by one. Only variables with *p* < 0.05 were considered for inclusion in the final multivariable model. The Hosmer-Lemeshow goodness-of-fit statistic was used to examine the performance of the final model. Receiver operating characteristic (ROC) curve analysis was performed to evaluate the performance of infertility duration and endometrial thickness in FET. The optimal cut-off points were determined using the Youden index. All statistical analyses were performed using SPSS statistics software (SPSS Inc., version 22.0; Chicago, IL, USA).

## Results

In this study, 1,803 embryos were transferred into 917 young women. The overall LBR was 53% (486/917), and the all twins birth rate was 36.8% (179/486). A total of 886 patients transferred two embryos, that the twins birth rate was 37.6% (179/476) after transfer of two frozen embryos. All first FET cycles were completed <1 year after cryopreservation.

The baseline maternal characteristics that were examined for their effects on the LBR after FET are presented in Table 1. According to our analysis, significant differences were found in infertility duration between the live-birth and non-live-birth groups (3.32 ± 2.00 years vs. 3.62 ± 2.13 years *p* = 0.027). The longer the infertility time was, the lower the LBR would be. Within the BMI < 25 kg/m^2^ and BMI ≥ 25 kg/m^2^ groups, there were no significant differences in BMI between the live-birth and non-live-birth subgroups. Regarding the other factors, the differences were not statistically significant between the live-birth group and non-live-birth group (Table 1).

**Table 1:** The baseline characteristics that may affect the LBR of FET cycles.

**Characteristics**	**Total**	**Live birth** **(*n* = 486)**	**Non-live birth (*n* = 431)**	***p*-values**
Age (years)	28.45 ± 3.00	28.33 ± 3.04	28.59 ± 2.95	0.185
BMI				0.376
<25 kg/m^2^ (%)	770 (84.0)	413 (85.0)	357 (82.8)	
≥25 kg/m^2^ (%)	147 (16.0)	73 (15.0)	74 (17.2)	
Duration of infertility (years)	3.46 ± 2.07	3.32 ± 2.00	3.62 ± 2.13	0.027
Indications for IVF-no. (%)				0.072
Tubal factor	561 (61.2)	281 (57.8)	280 (65.0)	
Male factor	239 (26.1)	135 (27.8)	104 (24.1)	
Combined factors	117 (12.8)	70 (14.4)	47 (10.9)	
Previous conception-no. (%)	304 (33.2)	163 (33.5)	141 (32.7)	0.791
Past-gynecology operation (%)	216 (23.6)	115 (23.7)	101 (23.4)	0.935
FSH (IU/L)	6.57 ± 1.63	6.56 ± 1.70	6.58 ± 1.55	0.849
LH(IU/L)	4.94 ± 1.92	5.03 ± 1.96	4.83 ± 1.87	0.128

*Unless otherwise indicated, values are presented as means ± SD for continuous variables and frequencies and percentages for categorical variables*.

*BMI, body-mass index; IVF, in vitro fertilization; FSH, follicle stimulating hormone; LH, luteinizing hormone*.

The embryological and clinical factors that may affect the LBR after FET cycles are shown in Table 2. According to the statistical analysis, there were no statistically significant differences in the cumulative gonadotropin (Gn) dose, number of two-pronuclear zygote (2PN) fertilized oocytes, number of cleavage-stage 2PN embryos or number of top-quality embryos transferred between the live-birth group and non-live-birth groups (Table 2). We found that the LBR of the natural cycles was higher than that of the artificial cycles, and that the difference was statistically significant (54.9 vs. 47.4% *p* = 0.048). A significant difference in endometrial thickness (1.01 ± 0.16 cm vs. 0.97 ± 0.18 cm, *p* = 0.003) was observed between the two groups. We observed a statistically significant difference in the LBR between FET with one frozen embryo and FET with two embryos. The LBR of the transfer of two frozen embryos was higher than that of a single transfer (53.3 vs. 32.3% *p* = 0.015).

**Table 2:** The embryological and clinical factors that may affect the live birth rate of FET cycles.

**Characteristics**	**Total**	**Live birth** **(*n* = 486)**	**Non-live birth (*n* = 431)**	***p*-values**
Endometrial thickness (cm)	0.99 ± 0.17	1.01 ± 0.16	0.97 ± 0.18	0.003
Cumulative Gn dose (IU)	1482.12 ± 443.86	1470.37 ± 443.26	1495.38 ± 444.67	0.395
No. of 2PN fertilized oocytes (*n*)	8.74 ± 4.37	8.91 ± 4.30	8.54 ± 4.45	0.203
No. of cleavage of 2PN (*n*)	8.58 ± 4.30	8.77 ± 4.23	8.37 ± 4.37	0.161
Endometrial preparation regimen -no. (%)				0.048
Natural	683 (74.5)	375 (77.2)	308 (71.5)	
Artificial	234 (25.5)	111 (22.8)	123 (28.5)	
No. of embryos transferred-no. (%)				0.019
One	31 (3.4)	10 (2.1)	21 (4.9)	
Two	886 (96.6)	476 (97.9)	410 (95.1)	
No. of top quality embryos transferred-no. (%)				0.152
Zero	9 (1.0)	3 (0.6)	6 (1.4)	
One	94 (10.3)	43 (8.8)	51 (11.8)	
Two	814 (88.8)	440 (90.5)	374 (86.8)	
Oocyte fertilization method-no. (%)				0.073
IVF	597 (65.1)	303 (62.3)	294 (68.2)	
ICSI	278 (30.3)	163 (33.5)	115 (26.7)	
Other protocols	42 (4.6)	20 (4.1)	22 (5.1)	

*Gn, gonadotropin; 2PN, two-pronuclear zygote; FET, frozen embryo transfer; IVF, in vitro fertilization; ICSI, intracytoplasmic sperm injection*.

The results of the univariate and multivariate logistic regression analyses of predictive factors for FET pregnancy outcome are summarized in Table 3. Finally, based on the univariate analysis results, infertility duration, endometrial thickness, endometrial preparation regimen for FET, timing of embryo transfer, number of embryos transferred, number of top-quality embryos, and oocyte fertilization method were included in the multivariable analysis. The Hosmer–Lemer goodness-of-fit test showed *p* = 0.313, which indicated that the data fit the model well. The multivariable logistic regression showed that infertility duration (OR: 0.933, 95% Cl: 0.876–0.995, *p* = 0.033), endometrial thickness (OR: 3.375, 95% Cl: 1.556–7.321 *p* = 0.002), and number of embryos transferred (OR: 2.653, 95% Cl: 1.226–5.743 *p* = 0.013) were the major factors affecting the “take-home-baby” rate of FET.

**Table 3:** Univariate and multivariate analysis of factors related to live birth in FET cycles.

	**Univariate analysis**			**Multivariate analysis**		
	**OR**	**95% CI**	***p*-values**	**OR**	**95% CI**	***p*-values**
Age (years)	0.971	0.930–1.014	0.185	Not selected	Not selected	Not selected
BMI			0.376	Not selected	Not selected	Not selected
<25 kg/m^2^ (%)	1.000	Ref				
≥25 kg/m^2^ (%)	0.853	0.599–1.214				
Duration of infertility (years)	0.932	0.875–0.992	0.028	0.933	0.876–0.995	0.033
Indications for IVF-no. (%)			0.073	–	–	–
Tubal factor	1.000	Ref				
Male factor	1.293	0.954–1.754				
Combined factors	1.484	0.990–2.225				
Previous conception-no. (%)	1.038	0.788–1.367	0.791	Not selected	Not selected	Not selected
Past-gynecology operation (%)	1.013	0.746–1.375	0.935	Not selected	Not selected	Not selected
FSH (IU/L)	0.992	0.916–1.075	0.849	Not selected	Not selected	Not selected
LH(IU/L)	1.054	0.985–1.129	0.128	–	–	–
Endometrial thickness (cm)	3.184	1.479–6.853	0.003	3.375	1.556–7.321	0.002
Cumulative Gn dose (IU)	1.000	1.000–1.000	0.394	Not selected	Not selected	Not selected
No. of 2PN fertilized oocytes	1.020	0.990–1.051	0.203	Not selected	Not selected	Not selected
No. of cleavage of 2PN	1.022	0.991–1.054	0.162	Not selected	Not selected	Not selected
Endometrial preparation regimen -no. (%)			0.049	–	–	–
Natural	1.000	Ref				
Artificial	0.741	0.550–0.998				
No. of embryos transferred-no. (%)			0.022	2.653	1.226–5.743	0.013
One	1.000	Ref				
Two	2.438	1.135–5.237				
No. of top quality embryos transferred-no. (%)			0.161	–	–	–
Zero	1.000	Ref				
One	1.686	0.398–7.146				
Two	2.353	0.584–9.473				
Oocyte fertilization method-no. (%)			0.074	–	–	–
IVF	1.000	Ref				
ICSI	1.375	1.032–1.834				
Other protocols	0.882	0.471–1.650				

*BMI, body-mass index; IVF, in vitro fertilization; FSH, follicle stimulating hormone; LH, luteinizing hormone; Gn, gonadotropin; 2PN, two-pronuclear zygote; FET, frozen embryo transfer; ICSI, intracytoplasmic sperm injection*.

*–, variable was removed in the final multivariable model*.

**Figure 1:**
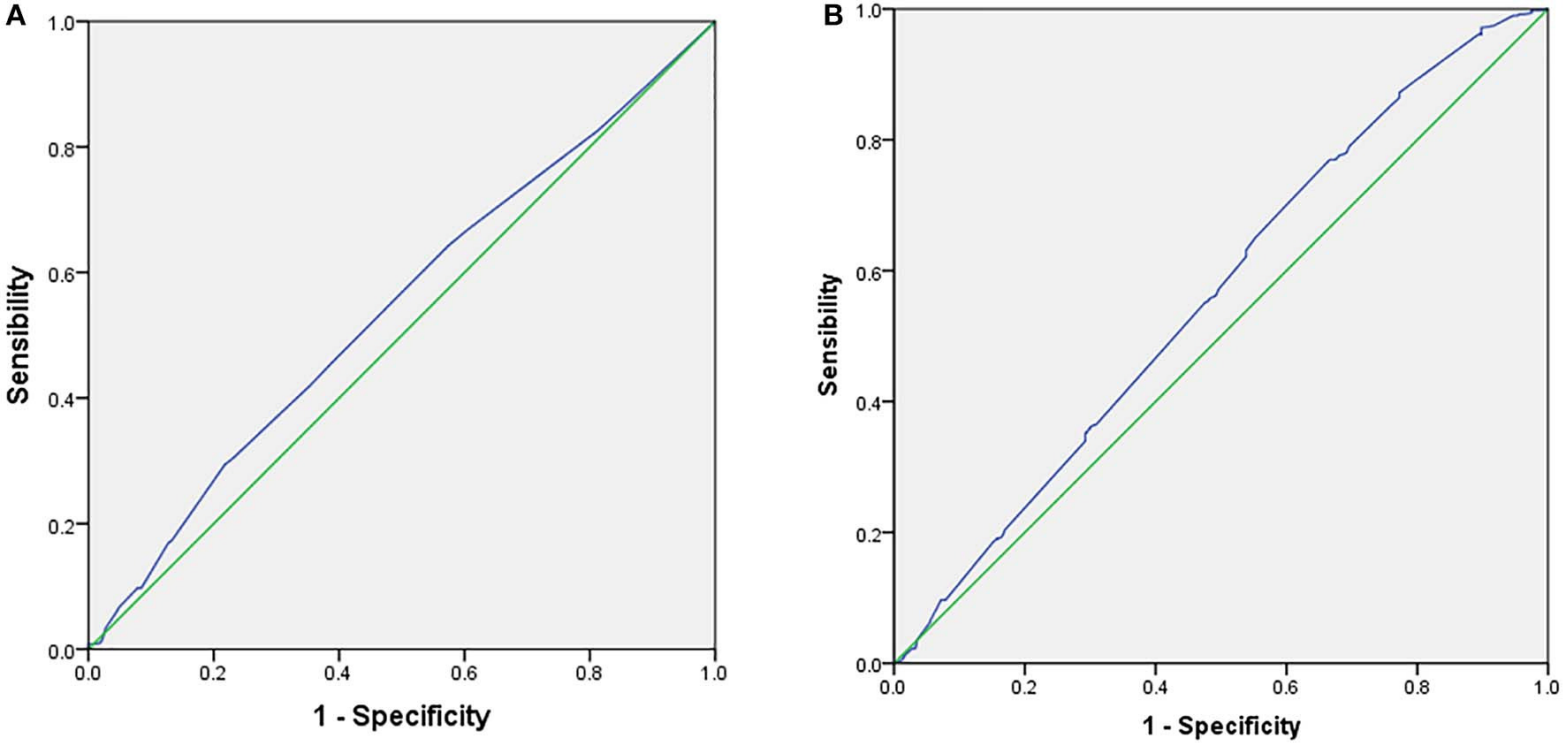
Receiver operator characteristic curve (ROC) for the predictive parameters of live birth rate in frozen embryo transfer. **(A)** ROC for duration of infertility as predictors of LBR in FET. **(B)** Receiver operator characteristic curve (ROC) for endometrial thickness as predictors of LBR in FET.

ROC curve analysis of infertility duration in FET and endometrial thickness in FET was performed for the predictive assessment of live birth (Figure 1). For infertility duration, the area under the curve (AUC) was 0.543. Youden's index for infertility duration was 0.07656, the cut-off point was 4.5 years, and the specificity and sensitivity were 29.47% and 78.19%, respectively. For endometrial thickness, the AUC was 0.562. Youden's index of endometrial thickness was 0.1037, the cut-off point was 0.89 cm, and the specificity and sensitivity were 33.41% and 76.95%, respectively.

## Discussion

This study assessed the LBR of 917 FET participants using a portion of the data from a large multicenter RCT. According to our study, in ovulating women, the infertility duration, endometrial thickness, and number of embryos transferred were important factors that affected the LBR after FET among young women.

Female age was a critical factor in predicting the LBR after FET. Recently, many large studies have shown that oocyte quality is poor in women who are older than 35 years and that this age group has lower ongoing pregnancy and clinical pregnancy rates than younger women (13–15). In this study, all included women were below 35 years of age. We still evaluated the effect of young age on the LBR, aiming to eliminate the effect of age on the pregnancy outcomes of women below 35 years of age. The results of this study showed no correlation between age and LBR among patients under 35 years of age.

There has been controversy among recent studies regarding whether an increase in BMI has an impact on the outcome of FET. Farhi et al. (16) found that the implantation and pregnancy rates of women with a BMI ≥ 25 kg/m^2^ were not significantly different from those of women with a BMI < 25 kg/m^2^ when high-quality embryos were transferred. Insogna et al. (17) studied 461 FET patients with good-quality day-5 or day-6 embryos following identical hormone replacement endometrial therapy. They found no statistically significant differences in the implantation rate or live-birth rate among underweight (<18.5 kg/m^2^), normal weight (18.5–24.9 kg/m^2^), overweight (25.0–29.9 kg/m^2^), and obese (≥30.0 kg/m^2^) women. In contrast, a retrospective study found that obesity was associated with declines in the clinical pregnancy rate and LBR (18). In our study, the number of patients with a BMI ≥ 30 kg/m^2^ was only 14, which was a small number of patients. Therefore, all patients were divided into two groups (BMI < 25 and ≥25.0 kg/m^2^). The results showed the absence of an association between the LBR and overweight. Whether a BMI ≥ 30 kg/m^2^ affects the LBR among young ovulating women still needs to be confirmed in a future study.

In this study, we found that prolonged infertility was associated with a decrease in the LBR after FET. Cai et al. (19) and Nelson et al. (20) reported that the infertility duration was a predictive factor of the FET outcome after IVF/ICSI. The pathogenesis of the adverse effect of prolonged infertility on pregnancy outcome remains unknown. However, this factor suggested that extending the waiting time for treatment during early infertility might hinder the outcome of assisted reproduction. This study showed that the cut-off point for infertility duration was 4.5 years, meaning that when the infertility time exceeded 4.5 years, the LBR after FET would be affected. Physicians need to advise women with long-term infertility to receive treatment by assisted reproductive technology and undergo FET as soon as possible.

Endometrial thickness is a crucial factor in determining the timing of FET. Bu et al. (21) found that endometrial thickness made a significant difference in the outcomes of FET cycles. El-Toukhy et al. (22) found significantly higher implantation and pregnancy rates for women with an endometrial thickness of 9–14 mm than in women with an endometrial thickness of 7–8 mm in exogenous hormone replacement cycles. In contrast, some studies found no correlation between FET cycle endometrial thickness and the LBR after FET (23, 24). Our results showed that endometrial thickness was one of the most important major predictive factors of a successful outcome following fresh transfers and FETs. The ROC curves showed that the cut-off point for endometrial thickness was 8.9 mm. This value is consistent with the clinical reality. Thus, when the endometrial thickness of a patient is ≥9 mm, the probability of a live birth is observed to be higher.

We observed that the LBR for natural cycles is higher than that for artificial cycles. However, we did not find a relationship between the LBR and the endometrial preparation regimen for FET after multivariate logistic regression analysis. Morozov et al. (25) showed that hormone replacement treatment for FET was associated with a lower endometrial thickness than that of natural cycles. Endometrial thickness is a major factor that can affect the LBR. Therefore, the two different endometrial preparation methods for FET might affect the FET outcome. Kawamura et al. (26) reported a prospective trial showing that natural cycles had a higher LBR than cycles with hormone replacement treatment for FET, but the difference was not statistically significant. A retrospective analysis showed that the natural cycle for the cleavage-stage frozen embryo was associated with a higher LBR than the cycle with hormone replacement treatment (27). Therefore, whether the two different endometrial preparation methods for FET affect the FET outcome remains unclear. Further research is necessary and warranted to clarify this issue.

In accordance with previous studies, the number of embryos, and number of top-quality embryos transferred affect the pregnancy outcome (28, 29). According to our results, only the number of frozen embryos transferred was an important clinical factor influencing LBR. The LBR associated with the transfer of two frozen embryos was higher than that associated with the transfer of one embryo. We did not find an association between the LBR and the number of top-quality frozen embryos transferred. However, these unrelated conclusions should be carefully considered because the original RCT protocol for the majority of FET cycles specified the selection of two top-quality D3 frozen-thawed embryos. Nevertheless, due to an insufficient number of embryos, poor embryo quality, or patient preference, a small proportion of patients had only one or zero D3 top-quality frozen-thawed embryo to transfer. Hence, further studies are needed to determine whether the timing of embryo transfer and the number of top-quality embryos transferred affect the LBR after FET.

The original data for this study were obtained from our previous RCT. All the patients were randomly assigned to the study, which should reduce the risk of bias. The relatively large sample size should lead to the most reliable and accurate results. To our knowledge, few studies addressing the factors affecting the LBR after FET, especially among young women, are available. Most publications report biochemical pregnancy or clinical pregnancy rather than live birth. We chose the LBR as the endpoint to better help guide clinical practice, and this is the first study to analyze the independent risk factors that could affect the LBR after FET in the young female population. There are also limitations in our study. The original data were from our previous RCT, in which the conditions for enrollment were strictly controlled. The original RCT selected a population of patients in whom two good embryos were transferred, which might lead to a high twin pregnancy rate. Therefore, it was difficult to represent the broader population. Thus, in the future, a large series of patients will be necessary to confirm the factors influencing the FET outcome.

## Data Availability Statement

The datasets generated for this study are available on request to the corresponding author.

## Ethics Statement

The studies involving human participants were reviewed and approved by Ethics Committee at Center for Reproductive Medicine, Shandong Provincial Hospital Affiliated to Shandong University. The patients/participants provided their written informed consent to participate in this study.

## Author Contributions

YS and Z-JC designed the trial and were in charge of the trial conduct. YP, GH, and YS designed the study. YS, HL, YP, GH, and ZW acquired the data. ZW, YP, and QW performed the statistical analyses. YP, HL, ZW, QJ, and QW interpreted the data. YP wrote the first draft of the report with inputs from GH, HL, YS, and Z-JC provided comments, participated in additional discussions, and revised the paper. All authors approved the final version.

### Conflict of Interest

The authors declare that the research was conducted in the absence of any commercial or financial relationships that could be construed as a potential conflict of interest.
