# Supplementary figures and images for: Major Factors Affecting the Live Birth Rate After Frozen Embryo Transfer Among Young Women
Source: Front Med (Lausanne). 2020 Mar 24;7:94. doi: 10.3389/fmed.2020.00094 (PMC7105776; doi:10.3389/fmed.2020.00094)

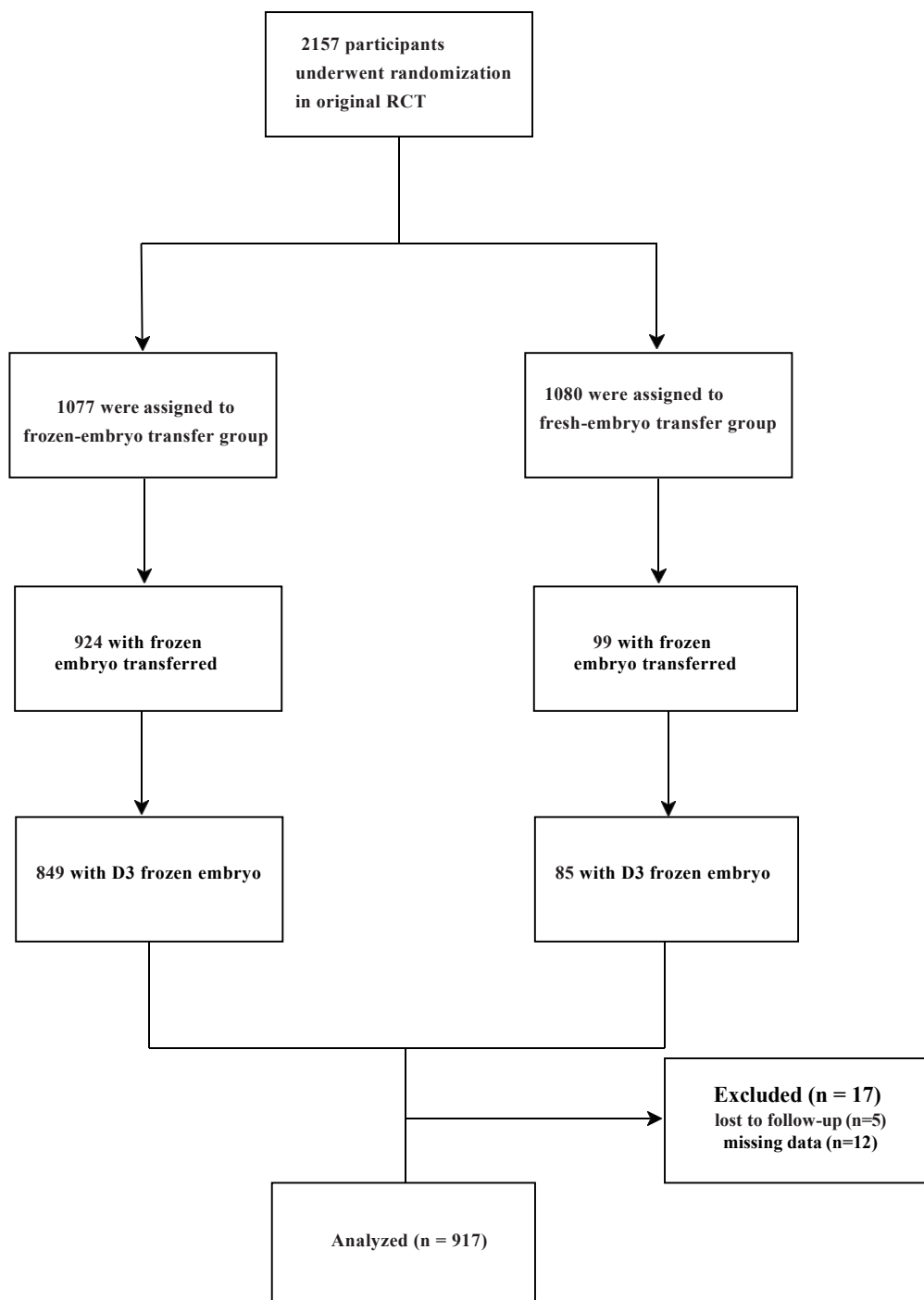

**Supplemental Figure 1.** Flow diagram.

Supplement: Supplementary file 1 [file Data_Sheet_1.PDF]
